# Chronic cigarette smoke exposure drives spiral ganglion neuron loss in mice

**DOI:** 10.1038/s41598-018-24166-9

**Published:** 2018-04-10

**Authors:** Stephen T. Paquette, Ryan P. Dawes, Isaac K. Sundar, Irfan Rahman, Edward B. Brown, Patricia M. White

**Affiliations:** 1 Department of Neuroscience, Ernest J. Del Monte Institute for Neuroscience, University of Rochester School of Medicine and Dentistry, Rochester, NY 14642 USA; 2 Department of Biomedical Engineering, Hajim School of Engineering and Applied Sciences, University of Rochester, Rochester, NY 14623 USA; 3 Department of Environmental Medicine, University of Rochester School of Medicine and Dentistry, Rochester, NY 14642 USA; 4 Present Address: Ortho Clinical Diagnostics, 513 Technology Blvd, Rochester, NY 14626 USA; 5 Present Address: Vertex Pharmaceuticals, 50 Northern Avenue, Boston, MA 02210 USA

## Abstract

Tobacco use is associated with an increased risk of hearing loss in older individuals, suggesting cigarette smoke (CS) exposure may target the peripheral auditory organs. However, the effects of CS exposure on general cochlear anatomy have not previously been explored. Here we compare control and chronic CS exposed cochleae from adult mice to assess changes in structure and cell survival. Two-photon imaging techniques, including the imaging of second harmonic generation (SHG) and two-photon excitation fluorescence (TPEF) from native molecules, were used to probe the whole cochlear organ for changes. We found evidence for fibrillar collagen accumulation in the spiral ganglion and organ of Corti, consistent with fibrosis. Quantitative TPEF indicated that basal CS-exposed spiral ganglion neurons experienced greater oxidative stress than control neurons, which was confirmed by histological staining for lipid peroxidation products. Cell counts confirmed that the CS-exposed spiral ganglion also contained fewer basal neurons. Taken together, these data support the premise that CS exposure induces oxidative stress in cochlear cells. They also indicate that two-photon techniques may screen cochlear tissues for oxidative stress.

## Introduction

Tobacco use correlates with an increased risk of high-frequency hearing loss in older individuals^1^. Since an estimated thirty-six million Americans smoke cigarettes^2^, this correlation confers a large social and economic burden. However, the origins of this correlation are unclear. In the United States, smokers are more likely to hail from poorer socioeconomic groups, who are at additional risk for hearing loss^3^. Moreover, individuals who accept the known health risks incurred through smoking may be less likely to protect their hearing from other sources of damage, including noise. These observations raise the possibility that smoking may not directly impact the cochlea, but rather correlate with other risk factors for hearing loss.

Burning tobacco initiates a complex chemistry of reactive organic molecules and free radicals, which are immediately inhaled into the lungs and sinuses of exposed individuals^4^. These molecules may access the cochlea through the bloodstream, or alternatively, via the Eustachian tube, which opens into the nasal part of the pharynx during swallowing^5^. One such component, nicotine, binds to nicotinic acetylcholine receptors (nAChR) present throughout the nervous system^6^, including the cochlea^7^. While the effects of CS exposure on inner ear cells have not been explored, there is epidemiological evidence that passive CS inhalation increases the likelihood of Eustachian tube dysfunction^8^. For endothelial cells and lung, CS exposure drives an inflammatory response as a consequence of oxidation^9^. Later stages of inflammation include cell death and destruction of the extracellular matrix (ECM, reviewed in^10^). The combination of apoptosis, inflammation, and ECM degradation causes alveolar enlargement and a loss of elasticity^11^. These destructive processes culminate into chronic obstructive pulmonary disease (COPD,^12^).

While the use of animal models with long-term CS exposure has contributed significantly to our current understanding of CS effects on the lung, little is known about CS effects on cochlear cells. Mice exposed to CS for six months in a pulmonary project also provided inner ear tissue for the current study, an additional anatomical analysis of CS-specific effects. This chronic CS exposure is sufficient to induce airspace enlargement, similar to emphysema, in exposed mice^13,14^. We hypothesized that, as in the lung, chronic CS exposure induces oxidative stress in the cochlea. We used two-photon microscopy to rapidly screen CS-exposed cochleae for altered regions. Alterations in collagen fiber microstructure, including some associated with fibrosis^15–17^, can be detected by imaging second harmonic generation (SHG) with specialized two-photon microscopy^18^. The infrared wavelengths deeply penetrate tissue without loss of resolution, and may be used to probe entire tissue samples in whole mount, enabling the unbiased identification of altered regions without the use of expensive reagents. Other biological materials associated with metabolism display intrinsic fluorescence that may be quantified with two-photon excitation fluorescence (TPEF). In this report, we used these imaging techniques together with an analysis of oxidative stress, neuronal survival, and efferent innervation to assess effects of chronic CS exposure on the cochleae from adult C57BL/6 J mice.

## Methods

### Animal usage

C57BL/6 J mice were purchased from the Jackson Laboratory (Bar Harbor, ME). Cochleae from air (n = 8) exposed mice and cigarette smoke (CS, n = 10) exposed mice were obtained for this study. Mice were fed on regular diet and water *ad libitum*. Both male and female mice were used in this study. All experiments were performed within compliance of the US Department of Health and Human Services and were approved by the University of Rochester Medical Center Committee on Animal Resources.

### Cigarette smoke (CS) exposure

Eight to ten-week-old mice were exposed to chronic treatment of CS using a Baumgartner-Jaeger CSM2082i cigarette smoking machine (CH Technologies, Westwood, NJ) for six months. Exposure was performed as previously described^19^. Treatments were performed at the Inhalation Core Facility at the University of Rochester. Mice were placed in individual wire cages housed in a closed plastic box which was connected to the smoking machine. CS was generated from 3R4F research cigarettes containing 10.9 mg of total particulate matter (TPM), 9.4 mg of tar, 0.726 mg of nicotine, and 11.9 mg of carbon monoxide per cigarette (University of Kentucky, Lexington, KY). Mice received a single 5 hour exposure once per day according to Federal Trade Commission protocol (1 puff/min of 2-s duration and 35 mL volume). Mainstream CS was diluted along with filtered air and directed into the exposure chamber. The noise level experienced by the mice during this procedure was measured at 70 dB SPL. Real-time monitoring of CS exposure was performed using a MicroDust Pro-aerosol monitor (Casella CEL, Bedford, UK) and verified daily by thermogravimetric sampling immediately after exposure was completed. CS concentration was adjusted to a constant level of ~300 mg/m^3^ TPM. Control mice received identical treatments, but with unlit cigarettes.

### Two-photon microscopy

Microdissected cochlear pieces were mapped using the MEEI ImageJ plug-in prior to imaging. TPEF imaging was performed using an Olympus Fluoview 1000 AOM-MPM system equipped with a DeepSee laser system. Samples were illuminated by a Spectra-Physics MaiTai HP DeepSee Ti:Sapphire laser, with the following characteristics: excitation/emission centered at 755/420–460 for NADH fluorescence, at 860/495–540 for FAD fluorescence, and at 800/495–540 for elastin fluorescence. Elastin parameters were used to find tissue regions. An Olympus 25×, 1.05 NA water immersion lens was used for all imaging. Single optical sections imaged with NADH and FAD parameters were used to generate heat maps of relative metabolic oxidation (n = 3 cochleae per condition, see^20,21^). Two 12-bit images from the same plane with NADH and FAD fluorescence were first added in ImageJ, and then the original FAD image was divided by the summed image (e.g., using the formula FAD/[FAD + NADH]). This 32-bit image was reduced to 8-bit and visualized with a heat-map ImageJ plugin^22^.

SHG and additional TPEF imaging was performed using a Fluoview FV300 scanning system on a BX61WI Olympus upright microscope (Olympus, Shinjuku, Tokyo) with incident light produced by a SpectraPhysics MaiTai Ti:Sapphire laser system. Incident 810 nm excitation light was circularly polarized using a New Focus Model 5540 Berek compensator (New Focus, Irvine, CA) and focused onto the sample stage using a water immersion lens (20×, 0.95 N.A.). Backward-scatter SHG and TPEF signals were then filtered from incident light using a 700 nm short-pass filter (E700SP-2P, Chroma). This would collect cellular fluorescence from FAD and NADH, extracellular fluorescence from elastin, and extracellular second harmonic generation (SHG) from fibrillar collagen. Forward-scatter SHG light was captured using an Olympus 0.9 N.A. optical condenser, reflected by a 475 nm long-pass dichroic mirror (475 DCSX, Chroma, Rockingham, VT), and filtered with a 405/30 nm band-pass filter (HQ405/30m-2P, Chroma, Rockingham, VT). Signals were collected by Hamamatsu HC125-02 photomultiplier tubes. Cochleae were documented for SHG at both the 16 and 32 kHz frequencies (n = 3 per condition).

### Immunostaining

Standard practices of decalcification and sucrose cryoprotection were used to protect the morphology of cochlear structures. Freshly dissected cochleae were immersed in 4% paraformaldehyde in PBS overnight, after removal of the stapes and puncture of the apical cochlear capsule. Cochleae were decalcified in 0.1 M EDTA at 4 °C on a rotating platform for 3–5 days, submersed in 30% sucrose, and embedded in OCT to present the 16 and 32 kHz regions in transverse sections. The tissue was snap-frozen in liquid nitrogen to preserve delicate structures, cryosectioned at 20 microns as serial sections, and dried onto Fisher SuperPlus slides. Sections were washed in PBS, blocked in PBS with 0.1% Triton and 5% donkey serum, and incubated overnight in primary antibody at 4 °C. Secondary antibody incubations were done at room temperature for one hour.

For whole mount staining, decalcified tissue was dissected as described^23^. Frequency mapping was done using an ImageJ plug-in developed by the Massachusetts Eye-Ear Institute’s Eaton-Peabody Laboratories (available at http://rsbweb.nih.gov/ij/). Dissected turns were washed in room temperature Dulbecco’s PBS (Gibco) and blocked for one hour in 1% Triton and 5% donkey serum in PBS. Primary antibody incubations of anti-synaptophysin (SYP) were performed at 37 °C for 20 hours, the tissue was washed in PBS, and secondary antibody incubation was performed at 37 °C for an additional 2 hours. Whole mounts were placed between two 50 mm coverslips in Fluoromount-G (Southern Biotech).

Primary antibodies were anti-βIII tubulin, clone TUJ1 (1:500, Millipore, RRID: AB_2210524), anti-3-nitrotyrosine (3NT) (1:200, Enzo Life Sciences, Inc., RRID: AB_10539899), anti-4-hydroxynonenol (4HNE) (1:100, Gene Tex, RRID: AB_424345) and anti-synaptophysin (SYP) (1:200, OriGene Technologies, RRID:AB_2623575). Secondary antibodies were purchased from Jackson Labs: donkey anti-goat Alexa 647 (1:500; RRID:AB_2340438), donkey anti-mouse Alexa 488 (1:500, RRID: AB_2340849), donkey anti-rabbit Alexa 594 (1:500, RRID: AB_2340622). Sections and whole mount tissue were imaged on an Olympus FV 1000 laser scanning confocal microscope. The optical stack depth used for SGN quantification was 6 microns, which allowed for restricted counting of a single SGN cell layer.

### Quantification and statistics

In this publication, spiral ganglion neurons were quantified in frozen sections, similar to^24–29^. Serial sections were obtained. Every other section was stained with anti-TUJ1 and DAPI to reveal neurons and nuclei respectively. Up to 5 sections were imaged for each turn, and only sections where the ganglion structure was intact, as assessed through bright-field images of these structures, were used in quantification. ImageJ 64 (NIH) was used to project optical stacks for quantification of spiral ganglion neurons. Photoshop 5.1 was used to generate masks to measure the area of Rosenthal’s canal. The area of these masks was measured using ImageJ 64. 4–6 cochleae per condition were processed for SGN quantification. 26 images of SGNs for each condition were blinded, randomized, and counted by a different individual. SGN density at the 16 kHz and 32 kHz turns was averaged for each cochlea, and these biological replicates were averaged. Statistical significance was determined by ANOVA and Student’s two-tailed t-test using Bonferonni’s adjustment in R (version 3.4.3).

For SYP quantification, CS-exposed and air-exposed cochleae were imaged at the 32 kHz region (n = 3 per condition). Confocal stacks were imported into the Amira FEI software suite with the XImagePAQ extension (RRID:SCR_007353). Deconvolution was performed using the iterative maximum likelihood estimation package. The stack was segmented and thresholded, and the Separate Objects function was used to reconstruct the individual SYP volumes. These were exported using the Connected Components function. Statistical significance was computed using the Wilcoxon rank sum test in R (version 3.4.3).

## Results

### Two-photon analysis reveals ECM remodeling and oxidative stress in CS-exposed cochleae

**Figure 1:**
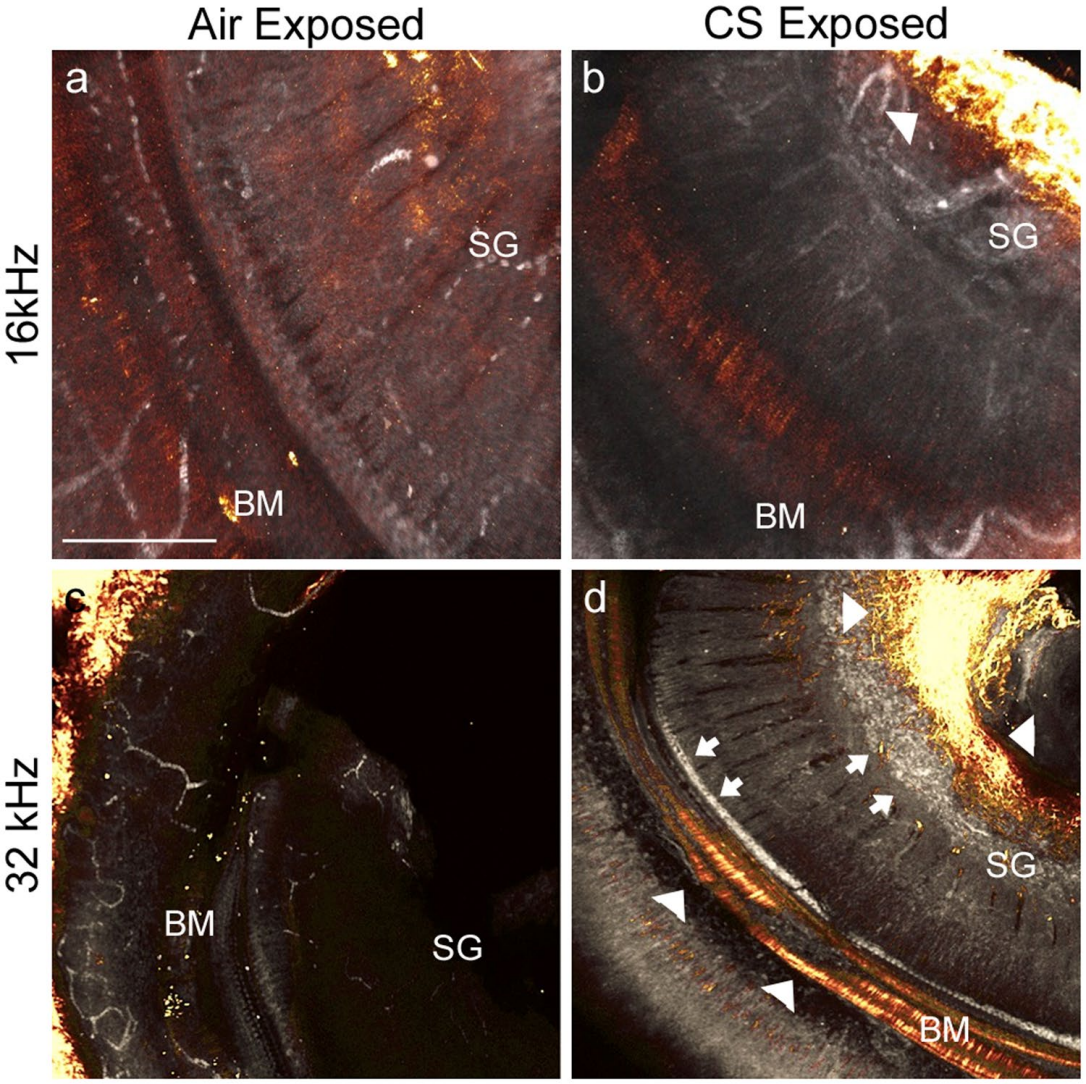
Extracellular matrix changes in the cochlea as a consequence of chronic CS exposure. (**a**) A whole mount preparation of the 16 kHz turn, from a mouse exposed to air, is imaged with second harmonic generation (SHG) to visualize fibrillar collagen (amber). Two-photon excitation fluorescence (TPEF) reveals intrinsic fluorescence from biomolecules such as elastin (gray). Scale bar: 200 microns. (**b**) A whole mount preparation of the 16 kHz turn, from a mouse exposed to CS, imaged with identical settings for SHG (amber) and TPEF (gray) as performed in (**a**). Arrowhead denotes changed SHG signal in spiral ganglion (SG). (**c**) A whole mount preparation of the 32 kHz turn from a mouse exposed to air, imaged for SHG (amber) and TPEF (gray). (**d**) A whole mount preparation of the 32 kHz turn from a mouse exposed to CS, imaged with identical settings for SHG (amber) and TPEF (gray) as performed in (**c**). White arrows highlight TPEF fluorescent accumulation in the spiral ganglion (SG) and inner hair cell region. White arrowheads (**b**,**d**) highlight SHG alterations in the medial SG and the basilar membrane (BM) of the organ of Corti. n = 3 cochleae per condition.

We used two-photon analysis to find regions of the mouse cochlea that may be altered as a consequence of chronic (6 month) CS exposure. Collagen remodeling is a signature of CS exposure in the lung^30^. We used SHG on whole mount pieces of cochleae to identify potential alterations in microstructural properties of collagen I, such as changes in interfibrillar spacing (Fig. 1, amber,^31^). Figure 1a is exposure-matched to 1b, and 1c is matched to 1d. At 16 kHz, the fields were comparable with the exception that increased SHG signal was observed in the CS-exposed spiral ganglion (SG; Fig. 1b, cf. 1a, amber, white arrowhead at SG). Similar signal was detected in the SG at 32 kHz (Fig. 1d, cf. 1c, amber, white arrowheads at SG). Signal was also detected in the basilar membrane at 32 kHz (Fig. 1d, amber, white arrowhead at BM). Elastin, FAD, and NADH display intrinsic fluorescence which may be detected through two-photon excitation fluorescence (TPEF)^32–34^. Novel fluorescent deposits (810 nm excitation, <700 nm emission) were observed in the CS-exposed SG and organ of Corti in the 32 kHz turn (Fig. 1d, gray, arrows). Little SHG or TPEF signals were observed in sensory regions of whole mount control cochleae (Fig. 1a,c, amber and gray), although some signal was observed in bony tissues lateral to and far from the sensory region (Fig. 1c, amber, left side of image). These data suggest that CS exposure may have effects on cochlear ECM in sensory regions.

**Figure 2:**
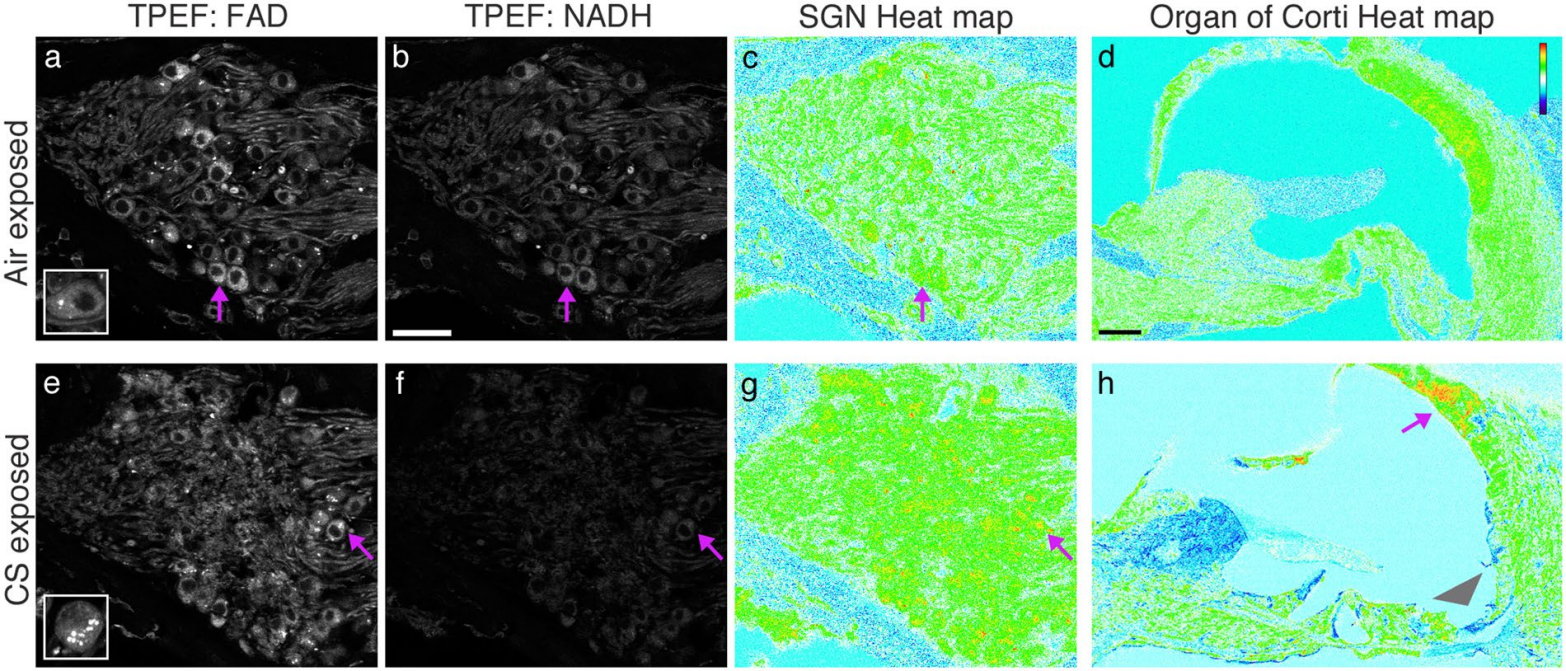
Quantitative TPEF to assess mitochondrial oxidation. (**a**) A single optical section from the 32 kHz spiral ganglion, from an air-exposed mouse, is imaged with TPEF filtered to collect FAD signal. A purple arrow highlights a neuron with a bright granular FAD-like signal. Inset shows a higher power confocal projection of a similar neuron. (**b**) Same section as in (**a**), imaged with TPEF filtered to collect NADH-like signal. NADH-like signal is not granular in aspect. Scale bar: 50 microns. (**c**) Heat map computed from (**a**) and (**b**) using the formula (FAD/(FAD + NADH)) as described in Methods. Red indicates regions of relatively higher FAD-like signal, indicating oxidative stress, whereas the cooler blue and white colors indicate regions of lower FAD-like signal. (**d**) Similar heat map computed with identical data from the apical organ of Corti region. Scale bar: 50 microns. (**e**) A single optical section from the 32 kHz spiral ganglion, from a CS-exposed mouse, is imaged with TPEF filtered to collect FAD signal. Exposure matches (**a**). Purple arrow highlights a neuron with bright granular FAD-like signal. Inset shows a higher power confocal projection of a similar neuron with many granules. (**f**) Same section as in (**a**), imaged with TPEF filtered to collect NADH signal. Exposure matches (**b**). No granules are observed. (**g**) Heat map computed from (**e**) and (**f**) reveals regions of increased oxidative stress in the spiral ganglion, indicated by red and orange speckles. (**h**) Similar heat map analysis for apical organ of Corti from CS-exposed mouse reveals cellular variation in oxidation. Gray arrowhead indicates the less oxidized spiral ligament, whereas the purple arrow indicates the more oxidized stria vascularis. n = 3 cochleae per condition.

**Figure 3:**
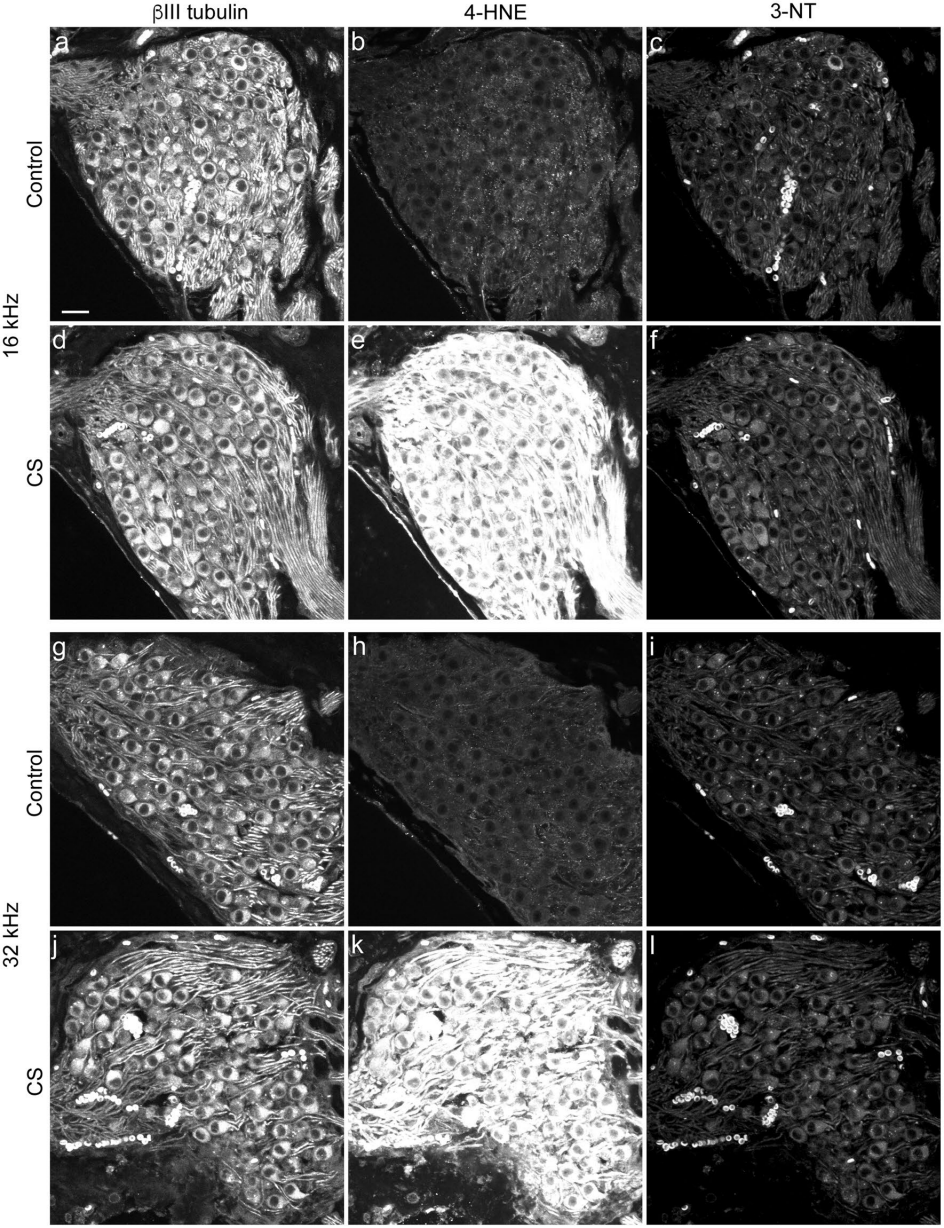
Oxidative stress of SGN as a consequence of chronic CS exposure. Sections through control (**a**–**c**, **g**–**i**) and CS-exposed (**d**–**f**, **j**–**l**) SGN are probed with antibodies to βIII tubulin (**a**,**d**,**g**,**j**) to reveal neurons, 4-hydroxy-2-nonenal (4-HNE, **b**,**e**,**h**,**k**) to reveal peroxidation, and 3-nitrotyrosine (3-NT, **c**,**f**,**i**,**l**) to reveal nitrosylation. Sections through the 16 kHz (**a**–**f**) and 32 kHz (**g**–**l**) are shown. n = 3 cochleae per condition. Scale bar: 20 microns.

Oxidative stress is a frequent consequence of CS exposure in the lung^35^. NADH is an electron donor, and FAD is an oxidized product that accumulates with mitochondrial oxidation. More stringent TPEF parameters may be used to quantify intrinsic fluorescence consistent with NADH or with FAD, and their relative levels may then be computed as a heat plot to identify regions of oxidation stress^20^. We performed this analysis on unstained cochlear sections (Fig. 2, n = 3 per condition, 32 kHz). SGNs from control cochleae emit fluorescence consistent with FAD (excitation/emission 860 nm/495–540 nm) and NADH (755 nm/420–460 nm) in the same imaging plane (Fig imaged for SHG (amber) and TPEF (gray. 2a,b). A heat map generated from these images reveals a few small, orange-red speckles, consistent with mitochondrial oxidation, among green and white cell bodies (Fig. 2c, purple arrows). Some speckles, originating from the FAD autofluorescence channel, are located within control SGNs and can be better appreciated in confocal projections (Fig. 2a, inset). CS-exposed cochlear sections from the same 32 kHz region display more and larger areas of orange-red speckles (Fig. 3e–g, purple arrow). The heat plot of CS-exposed SGNs displays less white and more green labeling, possibly because of lower overall NADH levels (cf. Fig. 2a,e and b,f). Moreover, confocal projections of individual CS-exposed neurons have more extensive FAD-like granulation (Fig. 2e, inset). Granulation is not observed in the NADH-like autofluorescence channel in either condition (Fig. 2b,f). These data suggest that basal SGNs experience oxidative stress from CS exposure. (Fig. 2a, cf. e).

In contrast to the SG, hair cells in the CS-exposed organ of Corti did not appear more oxidized in a similar heat plot representation (Fig. 2d, cf. with 2 h). We did, however, observe apparent oxidative stress in regions of the stria vascularis (Fig. 2h, purple arrow). Strikingly, the spiral ligament showed less oxidation in the CS-exposed organ of Corti compared to controls (Fig. 2h, grey arrowhead). These results indicate that oxidative stress in CS-exposed tissues are cell type specific.

### Oxidative stress and reduced SGN numbers in CS-exposed cochleae

To independently support the observation that SGNs are under oxidative stress from CS exposure, we tested an antibody to 4-hydroxy-2-nonenal (4-HNE), which binds to lipid peroxidation products, and an antibody to 3-nitrotyrosine (3NT), which detects accumulation of nitrated proteins, in sections of control and CS-exposed cochleae. Sections were counterstained for acetylated β-tubulin, which reveals neurons (Fig. 3a,d,g,j). Control SGN showed little evidence for either kind of damage (Fig. 3a–c,g–i), whereas the lipids in CS-exposed SGN were highly modified (Fig. 3e,k). In contrast, protein nitration was similar in both control and CS-exposed SGN (Fig. 3c,f,i,l), showing the specificity of lipid peroxidation. These data are consistent with our quantitative TPEF studies (Fig. 2), indicating that CS exposure increases oxidative stress in SGNs.

**Figure 4:**
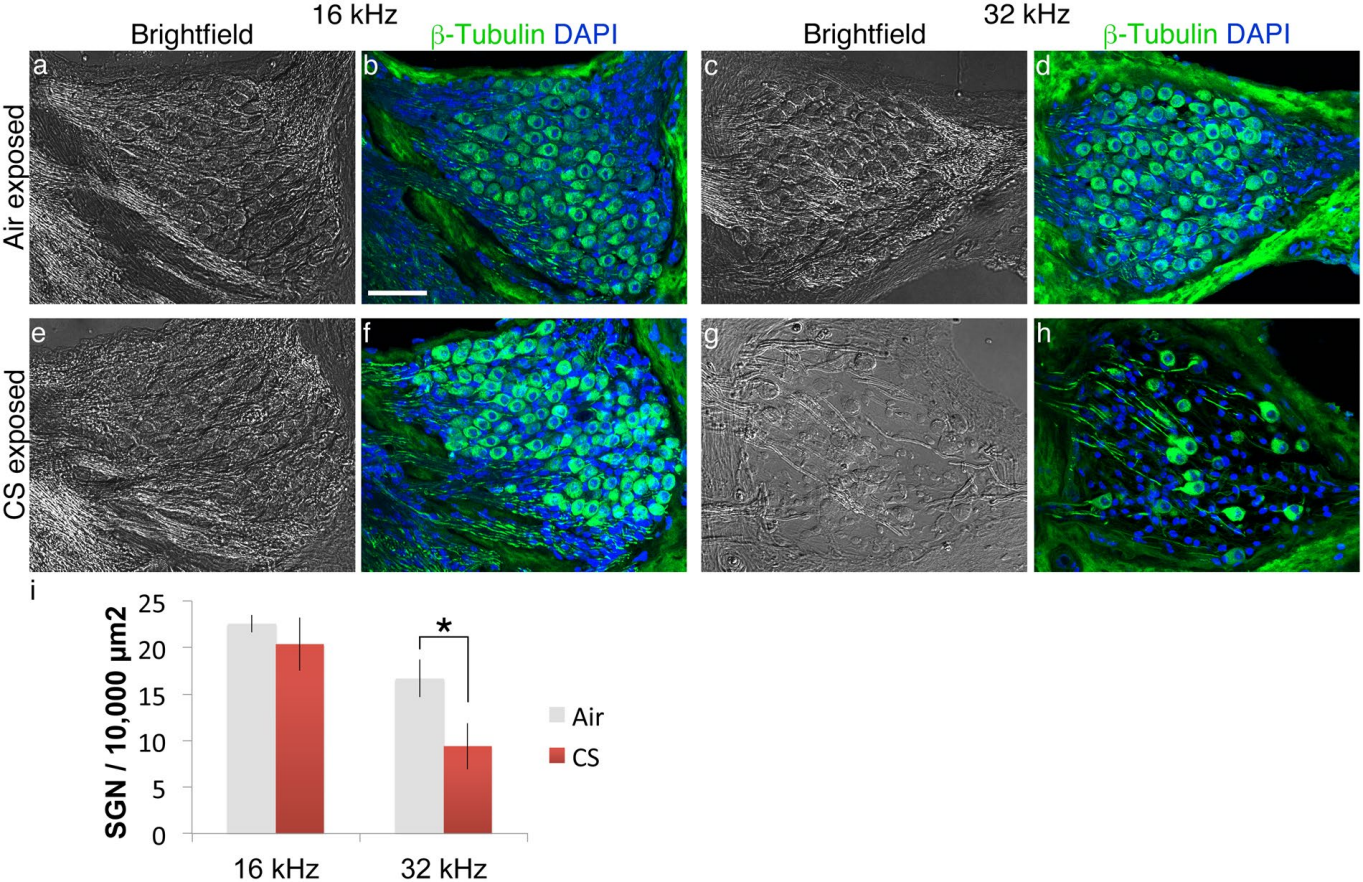
Basal SGN loss as a consequence of chronic CS exposure. (**a**) Brightfield image of representative section through the 16 kHz turn of an air-exposed mouse. (**b**) Same section as (**a**), probed with an antibody to acetylated β-tubulin (green) and DAPI (blue) to reveal neurons and cellular nuclei, respectively. Scale bar: 50 microns. (**c**,**d**) Similar images to (**a**,**b**), only from the 32 kHz turn of an air-exposed mouse. (**e**,**f**) Similar images to (**a**,**b**), only from the 16 kHz turn of a CS-exposed mouse. (**g**,**h**) Similar images to (**a**,**b**), only from the 32 kHz turn of a CS-exposed mouse. (**i**) Averages of biological replicates (+/− s.e.m.) quantified from sections indicate significantly fewer neurons in the CS-exposed high frequency regions (i, n = 4 or 5 cochleae per condition, ANOVA for condition, p = 0.03; Student’s t-test with Bonferroni adjustment for 32 kHz region only, p = 0.05).

Sections through the basal SG (Fig. 2) suggest that high-frequency SGNs may be lost in CS-exposed cochleae. Accordingly, we quantified SGNs per unit area on sections stained with the antibody to acetylated β-tubulin. Typical images are presented for both the 16 kHz (Fig. 4a,b,e,f) and the 32 kHz regions (Fig. 4b,c,g,h). The two conditions were significantly different (Fig. 4i, p = 0.03, n = 4, 6, CS- and air-exposed cochleae respectively, two-way ANOVA). The results for the 32 kHz region were significantly different (Fig. 4i, p = 0.05, Student’s two tailed t-test with Bonferoni adjustment). Taken together, these data confirm that CS exposure drives oxidative stress in SGN, and that CS-exposure correlates with reduced SGN numbers in the basal cochlea.

### Increased volumes of efferent projections to HCs in the CS-exposed cochlea

**Figure 5:**
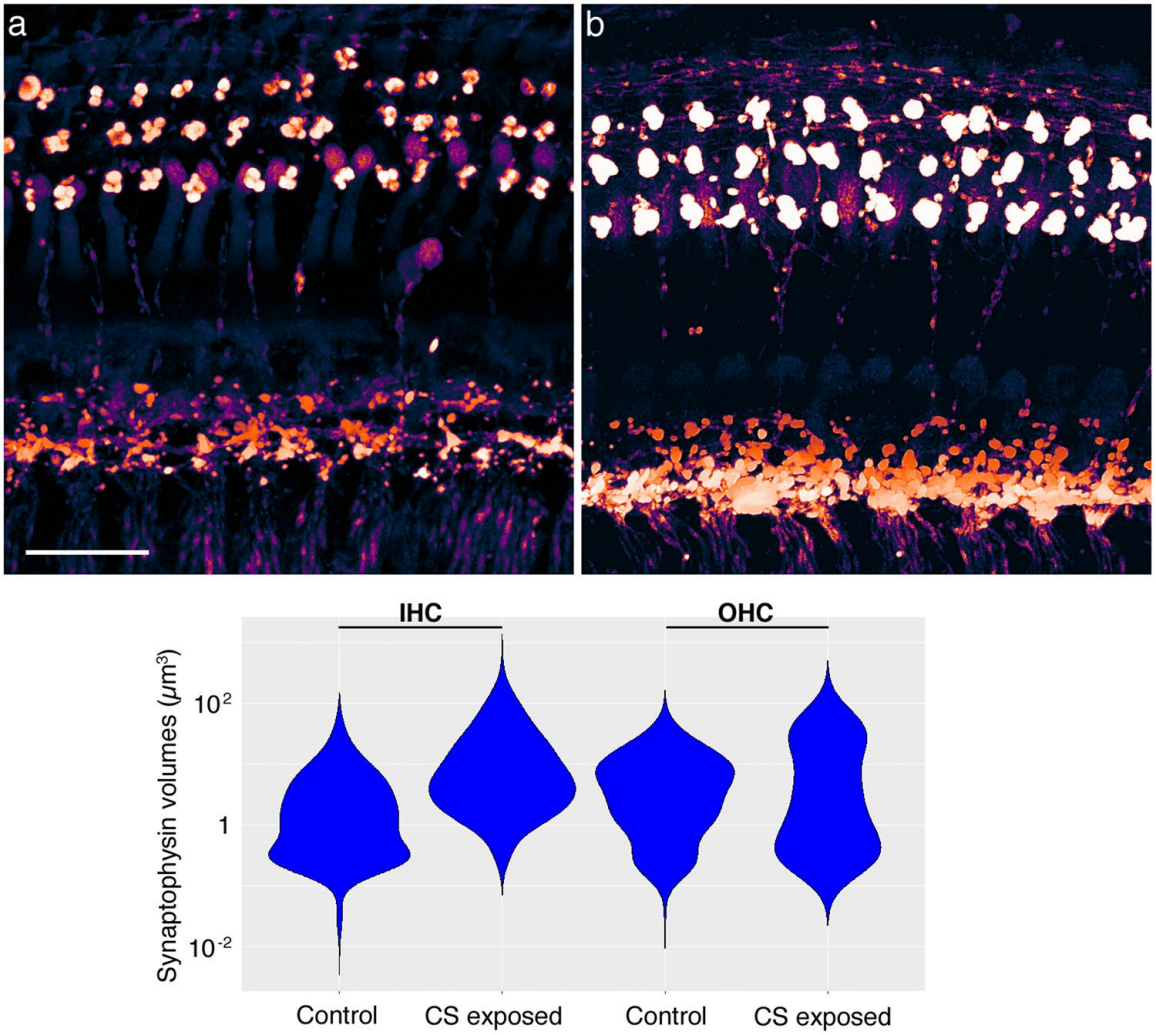
Enlarged efferent volumes in the basal cochlea as a consequence of chronic CS exposure. (**a**) A whole mount preparation of the 32 kHz turn from an air-exposed mouse, probed with an antibody to SYP (amber). This image derives from a confocal stack processed in Amira to detect edges. Scale bar: 20 microns. (**b**) A whole mount preparation of the 32 kHz turn from a CS-exposed mouse, similarly stained with anti-SYP (amber) and processed with edge-finding in Amira. (**c**) Quantification of SYP + volumes beneath IHCs (left pair) and beneath OHCs (right pair), contrasting air-exposed and CS-exposed samples as indicated. (n = 3 cochleae per condition, Wilcoxon rank sum test p < 2.2 × 10^−16^, both comparisons).

HCs express nicotinic acetylcholine receptors and their activities are modulated through cholinergic efferent signaling. We visualized efferent terminals in the 32 kHz region of air- and CS-exposed cochleae, using an antibody against synaptophysin (SYP). Three-dimensional modeling software was used to process the confocal images to obtain representations of separate efferent terminals (Fig. 5a,b, amber) and extract volumetric data (Fig. 5c). On average, efferent terminals were twice as large under CS-exposed OHCs (50.6 ± 7.9 µm^3^ for CS-exposed versus 26.6 ± 3.3 µm^3^ for air-exposed terminals). Efferent terminals were roughly eight times larger under CS-exposed IHCs (69.4 ± 12.1 µm^3^ for CS-exposed versus 8.3 ± 1.7 µm^3^ for air-exposed terminals). The distributions of these volumes were vastly different between conditions (Fig. 5c, n = 3 organs per condition, p < 2.2 × 10^−16^, Wilcoxon rank sum test, both for OHC terminals and IHC terminals). These data suggest that chronic CS exposure may be associated with alterations in cochlear efferent innervation.

## Discussion

This report describes for the first time histological changes in the mammalian cochlea after chronic exposure to tobacco smoke. Using two-photon imaging in mouse tissue, we find evidence for collagen alteration, as well as increased levels of FAD-like autofluorescence, in the high-frequency SG after six months of CS exposure. In other systems, similar findings correlate with increased fibrosis^15–17^ and mitochondrial oxidation^20^ respectively. To further support these findings, we analyzed levels of lipid peroxidation products in SGN after chronic CS exposure and found that they were greatly increased. We also report significant losses of 32 kHz SGN after CS exposure. Finally, we show enlargement of CNS efferent terminals beneath cochlear hair cells, suggesting that after chronic CS exposure, CNS modulation of auditory cells may also be altered. These findings indicate that chronic inhalation of tobacco smoke, a common environmental toxin, has specific and potentially deleterious effects on cochlear cells and structures.

SHG/TPEF imaging has been used to quantify fibrosis in liver tissue^17^ and kidney tissue^16^, and assess cartilage degeneration^36^ and tumor progression^31^. Others have previously used SHG and TPEF imaging to investigate pathological changes to the cochlea. In one such study, intravital multiphoton microscopy was used to measure the volume of the scala media compartment in the apical turns of control and pendrin-deficient mice^37^. In another, little difference was observed between the SHG signals detected from the SG and basilar membranes of control and noise-exposed cochleae^21^. In contrast, we see significant divergence between CS-exposed and control tissues (Fig. 1). The altered levels of SHG signal observed here may also be compared to similar changes in chronic arthritis models^38^. We anticipate that this rapid, non-destructive, and powerful imaging technique shows great promise for future studies that screen for widespread changes in the cochlea.

Increased oxidative stress, particularly in the form of lipid peroxidation products, is observed in many tissues after CS exposure, such as the lung^39^. We similarly see a dramatic and widespread increase in lipid peroxidation products in SGN after CS exposure (Fig. 3). This result supports our quantitative TPEF analysis of oxidative stress (Fig. 2). TPEF appears to be somewhat less sensitive for oxidative stress compared to antibodies for lipid peroxidation (Fig. 3). Lipid peroxidation may be due to the presence of various free radicals in tobacco smoke^4^. Non-nicotinic compounds in cigarettes activate antioxidant response genes in endothelial cells, correlating with CS extract toxicity for brain microvasculature^40^. Microvascular pathology is also associated with different forms of hearing loss^41^. Further experiments will be needed to determine if CS exposure drives a microvascular pathology in the inner ear.

It is also possible that chronic nicotine administration directly affects cochlear structures via the modulation of nicotinic acetylcholine receptors (nAChR). This idea is partly supported by our preliminary analysis of cholinergic efferent terminals, which are greatly enlarged in CS-exposed cochleae. Adult rodent SGNs express Chrna6, Chrna7, and Chrnb2^7^ and receive cholinergic input from neurons of the lateral olivocochlear (LOC) nucleus^42^ while OHCs express Chrna9 and Chrna10^43,44^ and receive cholinergic input from medial olivocochlear (MOC) neurons^42^. Stimulation of LOC neurons enhances the amplitudes of cochlear neuronal responses^45^, whereas ablation of LOC neurons depresses such amplitudes without affecting auditory thresholds^46^. In this way, the CNS may regulate a “set point” of excitability for SGNs, wherein cholinergic stimulation increases excitability^47^. In other systems, chronic nicotine administration can drive the up-regulation of functional nAChRs, especially those containing Chrnb2 subunits^6^. If a similar mechanism exists in the cochlea, we speculate that some SGNs in chronically CS-exposed mice could have a lower threshold for activation, driving oxidative stress. Notably, a study that compared hearing loss in hunters who either smoked or chewed tobacco found that both groups had greater hearing loss than hunters who did not do either, suggesting a specific role for nicotine^48^. Upregulation of Chrna9 and Chrn10 in OHCs might also explain the associated enlarged MOC terminals. Experiments that use only chronic nicotine administration will be helpful in dissecting its role in the cochlear changes described here.

This study may be considered opportunistic. Most studies of disease or environmental exposure concentrate on a single organ system. For example, the mice used here were part of a larger cohort from an investigation of COPD development in the mouse lung after chronic CS exposure. We note that in humans, chronic CS exposure is associated with a broad range of health problems in addition to COPD. Hearing loss, for example, has been linked to smoking in multiple studies^1,49–51^. Thus, CS exposure via systemic oxidative stress may have repercussions on hearing loss while COPD/emphysema ensues. However, the mechanism of CS-induced hearing loss is not known. Prior to commencing this secondary analysis, we discussed aspects of the protocol that were applicable to hearing. As the CS- and air-exposed mice experienced the same levels of environmental noise and stress, with the sole difference being the presence of CS, we considered that a secondary study, on whether CS induces anatomical changes in the inner ear, would be interpretable. However, opportunistic studies are unfortunately limited in scope, as we were unable to perform functional studies to assess if CS-exposed mice have changes in their hearing thresholds^50^ or distortion product emissions^49,52^, as reported for humans.

Future experiments could further confirm and extend the findings reported here. Functional hearing studies on CS-exposed animals, including studies of noise damage and recovery, will further illuminate the effects of smoking on the auditory system. It would be interesting to use SEM to examine the cochlear surface for changes in ECM, for example. Cochlear stiffness could also be assayed through cochlear gel analysis^53^. One may also wish to investigate potential changes in cortical auditory regions, such as the MOC and LOC. We have not yet determined which component of CS drives the changes we report, whether it is nicotine, oxidizing radicals or some combination of the two. Nonetheless, this is the first study to analyze the anatomical effects of chronic CS inhalation on the cochlea in an animal model. Our findings support an interpretation that exposure to CS increases oxidative stress in the cochlea and correlates with a reduction in the numbers of high-frequency spiral ganglion neurons.
